# Modulatory effects of traditional Chinese medicine and derived active metabolites on autoimmune-inflammatory signaling pathways in multiple sclerosis: a narrative review

**DOI:** 10.3389/fphar.2026.1907206

**Published:** 2026-09-01

**Authors:** Die Qian, Xiaohong Li, Jingjing Ge, Shuai Liu, Mingmin Na, Maokui Huang, Qingyan Mo, Mingqian Ju, Chunping Wan

**Affiliations:** 1 First Clinical Medical College, School of Pharmacy and School of Basic Medicine, Yunnan University of Traditional Chinese Medicine, Kunming, Yunnan, China; 2 The Fifth Affiliated Hospital of Yunnan University of traditional Chinese Medicine, Chuxiong, Yunnan, China

**Keywords:** autoimmune regulation, demyelination, multiple sclerosis, neuroinflammation, signal pathways, traditional Chinese medicine

## Abstract

Multiple sclerosis (MS), an immune-mediated neuroinflammatory disease, is characterized by demyelination, axonal injury and extensive destruction of the integrity of the central nervous system. Experimental autoimmune encephalomyelitis (EAE) is the most commonly used model for simulating the pathological features of human MS. Presently, there is still much room for improvement in the treatment strategy of MS. More and more studies have shown that traditional Chinese medicine (TCM) and related active metabolites are beneficial to the treatment of MS, and skillfully regulate the levels of inflammatory factors and immune system imbalance. Focusing on the core pathological mechanisms of MS, this review elucidates the beneficial effects of TCM in the prevention and treatment of MS *via* the modulation of autoimmune responses and inflammation-associated signaling pathways, as well as the underlying mechanisms of action. The main keywords “multiple sclerosis”, “experimental autoimmune encephalomyelitis”, “traditional Chinese medicine”, “natural metabolites”, “bioactive metabolites”, “autoimmune”, “inflammatory signals” and other details were retrieved from PubMed and Web of Science, Scopus, ScienceDirect, and Google Scholar. The results indicate that TCM preparations, including botanical drugs, active metabolite monomers and formulas, exert neuroimmunomodulatory effects *via* modulating Th1/Th2 and Th17/Treg cell balance and the CXCR6/CXCL16 signaling axis, and consequently inhibit excessive immune activation. Additionally, it regulates a variety of inflammation related signaling pathways, including NLRP3, TLR4/MyD88/NF-κB, MAPK, PI3K/Akt and JAK/STAT signal pathways, thus contributing to the treatment of MS. This study deepens our understanding of the mechanisms of TCM intervention in MS, and opens up a new perspective for clarifying the clinical value and development prospect of TCM in the treatment of MS.

## Graphical Abstract

**Figure:**
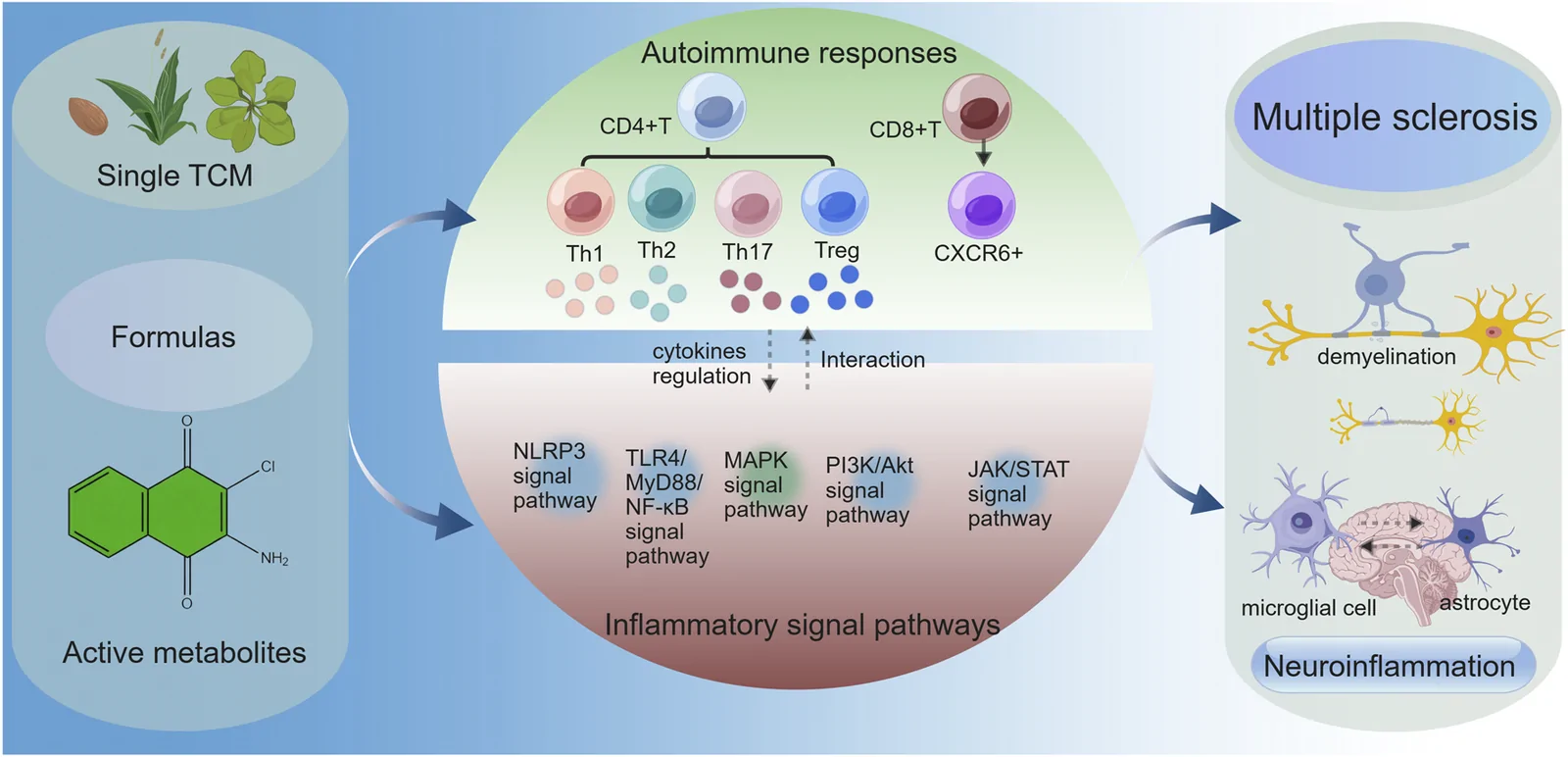

## 1 Introduction

Multiple sclerosis (MS) is a chronic, immune-mediated neuroinflammatory and neurodegenerative disease characterized by demyelination, axonal damage, and widespread disruption of the integrity of the central nervous system (Sharma, 2025). The latest epidemiological studies indicate that the estimated number of people with the disease worldwide exceeds 2.8 million, and the incidence of MS continues to rise (Portaccio et al., 2024). Clinically, MS patients typically exhibit focal or multifocal inflammatory demyelination of the central nervous system, accompanied by symptoms such as motor dysfunction, cognitive impairment, depression, fatigue, sensory dysfunction, and autonomic dysfunction, and may even experience permanent functional loss (Ahmad et al., 2025; Yu et al., 2025). At present, many people around the world are battling MS, making it a major public health challenge that causes immense suffering and imposes a heavy financial burden on patients (Portaccio et al., 2024).

Currently, patients with MS are categorized into distinct forms based on the severity and progression patterns of the disease: relapsing-remitting MS (RRMS), early primary progressive MS (PPMS), and secondary progressive MS (SPMS). The progression of the disease is generally associated with an increase in the rate of disability (Rashid et al., 2025). In the treatment of MS, Western medicine primarily focuses on measures to control acute flare-ups and slow the progression of the disease. During the acute phase, the administration of glucocorticoids, such as methylprednisolone, often helps protect the integrity of the blood-brain barrier (Rosenberg et al., 1996). In cases of severe MS relapse, plasma exchange (PLEX) is used clinically and provides some relief (Ikeda et al., 2015). For the long-term management of MS, disease-modifying therapies (DMTs) are the main therapeutic approach. They include injectable agents such as interferon-β, glatiramer acetate and monoclonal antibodies, as well as oral medications such as fingolimod, which effectively reduce relapse rates and slow disease progression (Hersh et al., 2024; Rashid et al., 2025). However, not all patients respond well to these treatments, and long-term use may lead to serious adverse reactions such as progressive multifocal leukoencephalopathy, infections, gastrointestinal reactions, and leukopenia, which significantly limit patients’ long-term treatment outcomes and quality of life (van Oosten et al., 1995). Consequently, the search for safe and effective adjunctive therapies has become a key focus of MS research.

Traditional Chinese medicine (TCM) has been used to treat illnesses in China for thousands of years (Qian et al., 2024; He et al., 2025). From the perspective of TCM, MS is believed to stem from congenital constitutional deficiencies, a deficiency of vital energy, invasion by pathogenic factors, depletion due to overwork, and dietary imbalances. A meta-analysis of 28 randomized controlled trials (RCTs) involving 1,971 participants showed that TCM combined with conventional treatment was significantly superior to conventional treatment alone in reducing Expanded Disability Status Scale (EDSS) scores and annual relapse rates (ARR), as well as in clinical symptoms. They were also well-tolerated, with no reports of serious adverse reactions (Guan et al., 2025). Therefore, as more and more people with MS benefit from TCM treatment, TCM is gaining greater recognition as a form of complementary and alternative medicine.

Although previous reviews have summarized potential therapeutic strategies for MS/EAE from the perspective of herbal therapies or naturally derived plant-based products, most studies have primarily categorized these strategies by herbal species, compound class, or therapeutic effect (Mojaverrostami et al., 2018; Guo et al., 2021). In contrast, the present review takes the regulation of autoimmune imbalance and inflammatory signaling pathways in MS/EAE by single botanical drugs, bioactive metabolites and prescriptions as the main thread (Table 1), aiming to provide a more focused immunological perspective for understanding the multi-component, multi-target and multi-pathway mechanisms by which traditional Chinese medicine intervenes in MS/EAE.

**TABLE 1:** Information on the sources and classification of TCM and active metabolites.

Classifications	Active metabolites/Chinese botanical drug	Scientific name	Chinese pharmacopoeia	Application site	References
Saponins	Ginsenoside Rd	*Panax ginseng* C. A. Mey or *Panax notoginseng* (Burkill) F.H.Chen	Ginseng radix Et rhizoma	Dry roots	(Zhu et al., 2014)
	Ginsenoside Re	*Panax ginseng* C. A. Mey or *Panax notoginseng* (Burkill) F.H.Chen	Ginseng radix Et rhizoma	Dry rhizomes	(Oh et al., 2024)
	Ginsenoside Rb3	*Panax ginseng* C. A. Mey or *Panax notoginseng* (Burkill) F.H.Chen	Ginseng radix Et rhizoma	Dry roots	(Zhang et al., 2025)
	Mogroside-V	*Siraitia grosvenorii* (Swingle) C.Jeffrey ex A.M.Lu and Zhi Y.Zhang	Siraitiae fructus	Dry fruits	(Xie et al., 2025)
	Astragaloside IV	*Astragalus mongholicus* Bunge	Astragali radix	Dry roots	(Liu et al., 2016; Yang et al., 2019)
	Anemoside A3	*Pulsatilla chinensis* (Bunge) Regel	Pulsatillae radix	Dry roots	(Ip et al., 2017)
Phenylethanoid glycoside	Forsythoside B	*Forsythia suspensa* (Thunb.) Vahl	Forsythiae fructus	Dry fruits	(Wang et al., 2025b)
Flavonoids	Baicalin	*Scutellaria baicalensis* Georgi	Sophorae flavescentis radix	Dry roots	(Zhang et al., 2015)
	Kurarinone	*Sophora flavescens* Aiton	Sophorae flavescentis radix	Dry roots	(Xie et al., 2018)
	Rutin	*Styphnolobium japonicum* (L.) Schott	Sophorae fructus	Dry flower buds and fruits	(Ge et al., 2025)
	Baicalein	*Scutellaria baicalensis* Georgi	Sophorae flavescentis radix	Dry roots	(Ying et al., 2023)
	Icariin	*Epimedium brevicornu* Maxim	Epimedii folium	Leaves and stems	(Gao et al., 2023)
	Scutellarin	Scutellaria baicalensis Georgi	Scutellariae radix	Leaves and stems	(Zhao et al., 2025)
	Luteolin	*Lagotis brachystachya* Maxim	N/A	Dry whole	(Tai et al., 2014)
	Total flavonoids of Astragus	*Astragalus mongholicus* Bunge	Astragali radix	Dry roots	(Han et al., 2023)
Isoflavones	Formononetin	*Astragalus mongholicus* Bunge	Astragali radix	Dry roots	(Fu et al., 2023)
Alkaloids	Berberine	*Coptis teeta* Wall	Coptidis rhizoma	Dried stems	(Qin et al., 2010)
	Sinomenine	*Sinomenium acutum* (Thunb.) Rehder and E.H.Wilson	Sinomenii caulis	Dried roots and stems	(Kiasalari et al., 2021)
	Matrine	*Sophora flavescens* Aiton	Sophorae flavescentis radix	Dry roots	(Liu et al., 2017)
Lignans	Arctigenin	*Arctium lappa* L	Arctii fructus	Dry fruits	(Li et al., 2016)
	Magnolol	*Magnolia officinalis* Rehder and E.H.Wilson	Magnoliae officinalis cortex	Barks	(Chen et al., 2023)
Terpenoids	Celastrol	*Tripterygium wilfordii* Hook.f.	N/A	Dry velamen	(Wang et al., 2015)
	Tripterygium glycosides	*Tripterygium wilfordii* Hook.f.	N/A	Dry roots	(Qiu et al., 2017)
	Eriocalyxin B	*Isodon eriocalyx* (Dunn) Kudô	N/A	Whole plants	(Lu et al., 2013)
	Kirenol	*Siegesbeckia orientalis* L.	Siegesbeckiae herba	Dry the above-ground parts or fruits	(Xiao et al., 2015)
	Ginkgolide K	*Ginkgo biloba* L.	Ginkgo folium	Leaves and seeds	(Yu et al., 2019)
	Ginkgolide B	*Ginkgo biloba* L.	Ginkgo folium	Leaves and seeds	(Yin et al., 2019)
	Artemisinin	*Artemisia annua* L.	Artemisiae annuae herba	Aboveground parts	(Gao et al., 2020)
	Cornel iridoid glycoside	*Cornus officinalis* Siebold and Zucc.	Corni fructus	Dry fruits	(Yin et al., 2014; Qu et al., 2019)
	β-elemene	*Curcuma aromatica* Salisb.	Curcumae rhizoma	Dry rhizomes or essential oil	(Zhang et al., 2011)
Coumarin derivatives	Daphnetin	*Daphne odora* Thunb.	N/A	Dry roots or tubers	(Wang et al., 2016)
Polyphenols	Curcumin	*Curcuma longa* L.	Curcumae longae rhizoma	Dry rhizomes	(Dolati et al., 2018)
	Gastrodin	*Gastrodia elata* Blume	Gastrodiae rhizoma	Dry rhizomes	(Shi et al., 2025)
	Epigallocatechin-gallate	*Camellia sinensis* (L.) Kuntze	N/A	Dry leaves	(Lovera et al., 2015)
Anthraquinones	Emodin	*Rheum palmatum* L. or *Rheum tanguticum* (Maxim. ex Regel) Balf. or *Rheum officinale* Baill.	Rhei radix Et rhizoma	Dry roots or rhizomes	(Zheng et al., 2022)
Naphthoquinones	Plumbagin	*Plumbago zeylanica* L.	N/A	Dry roots	(Zhang et al., 2014)
Benzoquinones	Embelin	*Embelia ribes* Burm. f.	N/A	Dry fruits	(Anika et al., 2024)
Polysaccharides	Astragalus polysaccharide	*Astragalus mongholicus* Bunge	Astragali radix	Dry roots	(Zhao et al., 2024)
Phytosteroid	Guggulsterone	*Commiphora wightii* (Arn.) Bhandari	Myrrha	Oleo-gum resin	(Kumar et al., 2021)
Monoterpene glycoside	Paeoniflorin	*Paeonia lactiflora* Pall.	Paeoniae radix rubra	Dry roots	(Zhang et al., 2017)
Chinese botanical drugs	Frankincense	*Boswellia sacra* Flück.	Olibanum	Aromatic oil-containing resin	(Stürner et al., 2018)
	Zingiber officinale Roscoe	*Zingiber officinale* Roscoe	Zingiberis rhizoma	Fresh rhizomes	(Borgonetti et al., 2025)
	Rhodiola rosea	*Rhodiola rosea* L.	Rhodiolae crenulatae radix Et rhizoma	Dry roots and rhizomes	(Lin et al., 2020)
	Alpinia oxyphylla fruit	*Alpinia oxyphylla* Miq.	N/A	Dry fruits	(Huang et al., 2019)
	Rhizomes of Panax japonicus	*Panax japonicus* C. A. Mey.	Panacis japonici rhizoma	Dry rhizomes	(Wang et al., 2023a)
	Radix Rehmanniae	*Rehmannia glutinosa* (Gaertn.) Libosch. ex DC.	Rehmanniae radix	Fresh or dried tubers	(Li et al., 2018)
	American ginseng	*Panax quinquefolium* L.	Panacis quinquefolii radix	Dry roots	(Szczuka et al., 2019)

## 2 Methods-literature search strategy

A systematic literature search was conducted in PubMed, Web of Science, Scopus, ScienceDirect, and Google Scholar up to July 2026. The following subject terms were used for the search: “Multiple sclerosis” OR “experimental autoimmune encephalomyelitis” OR “EAE model” AND “traditional Chinese medicine” OR “extract” OR “formula” OR “prescription” OR “bioactive metabolites” AND “autoimmune” OR “inflammatory signals”, and other details that may need to be added. In parallel, ethnopharmacological records were retrieved from authoritative traditional medicine sources, like *the Pharmacopoeia of the People’s Republic of China*. Eligible studies include original *in vivo*, *in vitro*, and clinical studies that report on the effects of TCM, active metabolites, or formulas in modulating autoimmune and inflammatory signaling pathways to intervene in MS/EAE. The reference lists of the existing reviews were read and the eligible studies were screened. The exclusion criteria were studies that did not address immune-inflammatory mechanisms in treatment and studies that consisted solely of clinical observations without any discussion of underlying mechanisms. Filter by keywords and summarize the relevant research literature. All references were manually organized and categorized according to the thematic framework and incorporated into the relevant sections of the text for discussion and analysis. All cited pharmacological studies involving botanical drug or species name were taxonomically validated using the Medicinal Plant Names Services (MPNS) portal.

## 3 The effects of TCM on autoimmune in pathological progression of MS

### 3.1 Th1/Th2 and Th17/Treg cells

T helper cells (Th) 1/Th2 are a type of cell that mediates cellular immunity and differentiates from CD4^+^ T cells, playing a significant role in the regulation of MS (Hedegaard et al., 2008). Th1 and Th2 have different functions, which are attributed to the secretion of different cytokines. Among them, Th1 cells secrete cytokines such as IL-2, IL-3, IFN-γ, and TNF-α, which promote inflammation in various diseases, including MS (Gor et al., 2003; Khademi et al., 2004). In contrast, Th2 cells secrete IL-4, IL-5, IL-6, IL-10, IL-13, and TGF-β, exerting effects opposite to those of Th1 cells and suppressing Th1 proliferation (Gor et al., 2003; Hedegaard et al., 2008). Optimally regulated Th1/Th2 cellular subset immune balance is essential for maintaining systemic immunological homeostasis and represents a pivotal therapeutic target in multiple immune-mediated diseases.

T helper (Th) 17 cells express the RAR-related orphan receptor γt (RORγt) transcription factor and secrete cytokines such as IL-17, IL-6, IL-21, and IL-22, thereby promoting inflammatory responses in tissues (Kuwabara et al., 2017). To date, six isoforms of IL-17 have been identified, with IL-17A attracting considerable attention. It can induce the production of GM-CSF, CXCL1, and CXCL2, and acts in synergy with cytokines TNF-α, IL-6, and IL-1β to increase cellular inflammation in MS (Kuwabara et al., 2017). In fact, the presence of Th17 cells is not pathogenic in itself, but when driven by IL-1β and IL-23 synergistically stimulates the proliferation of mouse splenocytes and induces IL-17 production, thereby increasing the incidence and severity of MS (Li et al., 2011).

The proper functioning of regulatory T cells (Tregs) is crucial for the onset and progression of autoimmune diseases. Research has found that the isolation of dysfunctional Tregs from MS patients serves to confirm this view (Dyczko et al., 2025). Foxp3, a forkhead/winged helix transcription factor, is a key regulatory factor for Tregs and, together with CD4 and CD25, constitutes the surface markers of Tregs. Studies have shown that the cytokines TGF-β, IL-2, and IL-35 promote Foxp3 expression, stimulate Tregs to produce the immunosuppressive cytokine IL-10, and thereby suppress excessive immune responses (Zong et al., 2024). Conversely, the inflammatory cytokines TNF-α and IL-6 promote the expression of functionally suppressed Tregs (Zong et al., 2024). In the CNS of EAE mice, Treg cells are disrupted by certain genetic signals and converted into pathogenic Th17 cells (Benamar et al., 2025).

Accumulating evidence has demonstrated that a wide range of botanical extracts, purified metabolites and TCMs exert therapeutic effects against MS mainly through modulating the balance of Th17/Treg cells. Astragalus polysaccharide (APS), is a class of water-soluble polysaccharides that exhibits various biological activities related to autoimmune diseases, cancer, cardiovascular diseases, and other conditions. The latest research showed that APS inhibits the progression of EAE by regulating the Th17/Treg balance in the CNS and peripheral blood (Ma et al., 2025). Ginkgolide K, a diterpene derived from the botanical drug *Ginkgo biloba* L., was reported by Yu et al. (2019) that after interfering with EAE mice, it could increase the proportion of Tregs, reduce the proportion of Th17, inhibit the expression of chemokine molecules CCL-2, CCL-3 and CCL-5, and limit the migration of inflammatory cells to the spinal cord. Another similar finding was reported by Zhang et al. (2011), who treated EAE mice with the sesquiterpene β-elemene. They indicated that it alleviated EAE symptoms by inhibiting the differentiation and development of Th17 cells, downregulating the expression of IL-6, IL-23, and RORγt signaling, and promoting the expansion of Treg cells. In addition, a large body of research has shown that Th1 and Th17 cells have attracted significant attention in the context of autoimmune diseases. The effective metabolites derived from TCM, such as baicalin (Zhang et al., 2015), kurarinone (Xie et al., 2018), berberine (Qin et al., 2010), eriocalyxin B (Lu et al., 2013), daphnetin (Wang et al., 2016) and anemoside A3 (Ip et al., 2017), all can inhibit the differentiation of Th1 and Th17 cells, thereby improving the progression of MS. However, the effects of these metabolites are also diverse. It is particularly important to note that berberine does not affect the number of CD4^+^ Foxp3^+^ Treg cells. Subsequent studies reached different conclusions that administration of berberine at doses of 30 mg/kg and 60 mg/kg to EAE mice promoted the proliferation of Th2 and Treg cells and delayed disease progression. Paeoniflorin, a core active metabolite in *Paeonia lactiflora* Pall., reduces the number of Th17 cells in the CNS and spleen of EAE mice, rather than directly inhibiting the differentiation of Th17 cells (Zhang et al., 2017). Research has found that the active metabolite kirenol reduces the proportion of Th1 and Th17 cells in the lymph nodes of EAE mice (Xiao et al., 2015). Plumbagin restricted the expression of Th1- and Th17-polarized cytokines in mouse dendritic cells (Zhang et al., 2014). Similarly, arctigenin, an effective metabolite extracted from *Arctium lappa L.*, has a wide range of pharmacological effects (Guo S. et al., 2020; Chang et al., 2021). Experimental studies *in vivo* and *in vitro* showed that this metabolite can inhibit the expression of IFN-γ, transcription factors T-bet, IL-17A, IL-17F and ROR-γt (Li et al., 2016). Zhu et al. (2014) reported an interesting study in which ginsenoside Rd, a bioactive metabolite derived from *Panax ginseng* C.A. Mey., was used to treat EAE mice. It has the ability to reduce the permeability of the BBB, regulate the secretion of IFN-γ and IL-4, and promote the transshipment of Th2 in the supernatant of cerebral cortex and spleen cell culture, thereby alleviating the clinical symptoms of EAE.

A phase II clinical trial found that frankincense extract significantly reduced the levels of cytokines TNF-β, IL-4, IL-5, GM-CSF, IL-17A, IL-2, and IFN-γ, while increasing the proportion of Treg cells and the levels of IL-10 secreted by CD8^+^ T cells, thereby improving the severity of brain parenchymal lesions and reducing the volume of brain atrophy in RRMS patients (Stürner et al., 2018). In another clinical trial, crocin capsules significantly reduced serum levels of TNF-α and IL-17 in patients with MS (Ghiasian et al., 2019). Saffron syrup was shown to alleviate fatigue in these patients (Ashtiani et al., 2020). Although these botanical medicines have demonstrated good biological activity in clinical settings, these studies did not delve deeply into immunological targets. Zingiber officinale Roscoe was administered to EAE mice, and the results indicated that the drug reduced BBB permeability and decreased the production of the cytokines IL-6 and IL-17, thereby alleviating the amplification of the inflammatory response (Borgonetti et al., 2025). Rhodiola rosea, a Qi-invigorating medicine, has the ability to suppress Th1 and Th17 cells and restore Treg cells, and it maintains the stability of the CNS and autoimmune system microenvironments by regulating the Th17/Th1, Th17/Th2, and Th17/Treg ratios in the tissues of EAE mice (Lin et al., 2020). Huang et al. (2019) studied Alpinia oxyphylla Fruit, a botanical drug used both as food and for medicinal purposes. They found that this botanical drug could downregulate the levels of T-bet, ROR-γt, IFN-γ and IL-17 in the spinal cord of EAE mice, regulate the Th1/Th17 immune response, and thereby alleviate the inflammatory response and nerve damage of EAE.

There is much evidence to support the compatibility of TCM prescriptions for the treatment of MS. Sheng Jiang powder, formulated with *Rheum officinale* Baill., *Curcuma longa* L., *Bombyx mori* Linnaeus and *Cryptotympana pustulata* Fabricius, exhibits the ability to restore the peripheral Th17/Treg immune imbalance in MOG_35-55_-induced EAE mice and increase the level of anti-inflammatory factors to alleviate the clinical symptoms of EAE (Wu et al., 2025). Huangqi-Guizhi-Wuwu decoction, as recorded in the *Synopsis of the Golden Chamber*, has the effects of regulating qi and warming the meridians, promoting blood circulation to relieve bi syndrome, and is widely used in clinical practice to treat neurological disorders. It is formulated with *Astragali Radix*, *Cinnamomi Ramulus*, *Paeoniae Radix Alba*, *Zingiberis Rhizoma Recens* and *Jujubae Fructus* to produce a synergistic effect. Xu et al. (2024) demonstrated that Huangqi-Guizhi-Wuwu decoction could increase the proportion of Treg cells in EAE mice, inhibit the proportion of Th1 and Th17 cells, and prove this result by detecting the characteristic cytokines, namely, up-regulating the secretion of IL-10 and the level of Foxp3, and down-regulating the expression of IFN-γ, IL-17, ROR-γT and T-bet. Qiu et al. (2018) found that Pien Tze Huang alleviated the recurrence and remission of EAE mice by reducing the percentage of Th1 and Th17 cells. Similarly, Bu Shen Yi Sui capsule was composed of *Rehmanniae Radix*, *Rehmanniae Radix* Praeparata, *Polygoni Multiflori Radix*, *Rhei Radix et Rhizoma*, *Leonuri Herba*, *Fritillariae Thunbergii Bulbus*, *Hirudo*, *Scorpio* (scorpion), *Gastrodiae Rhizoma*, and *Forsythiae Fructus*. The study reported that this formula exerts a neuroprotective effect in EAE mice by regulating the Th17/Treg ratio and downregulating the expression of IL-17A, IL-6, IL-23, TGF-β1, and Foxp3 (Zheng et al., 2015). Based on current preclinical evidence, several classical formulas and Chinese patent medicines may exert anti-inflammatory and immunomodulatory effects at the formula level, potentially involving multi-component and multi-target interactions. These findings may provide mechanistic insights into their possible relevance to MS.

### 3.2 CXCR6/CXCR16 signal axis

CXC chemokine receptor 6 (CXCR6), also known as Bonzo and STRL33, is a G protein-coupled receptor with seven transmembrane domains (Li et al., 2022; Bao et al., 2023). C-X-C motif ligand 16 (CXCL16) is a chemokine classified as a member of the ELR-CXC subfamily and specifically binds to CXCR6, playing a critical role (Bao et al., 2023). Currently, there has been evidence indicating that CXCR6 is the most pathogenic CD4^+^ T cell-specific surface marker, especially pathogenic Th17 cells (Hou and Yuki, 2022). Interestingly, CXCR6 and CXCL16 accumulate in large quantities in the brain, both before and after the onset of acute EAE. This may be because neutrophils expressing CXCR6 migrate to the brain following antigen presentation, where they interact with Th17 cells, inducing neuroinflammation and leading to axonal damage and demyelination (Wojkowska et al., 2014). Therefore, CXCR6/CXCL16 plays a regulatory role in the adhesion, migration, and cytotoxicity of immune cells, making it a key factor driving the progressive course of MS (Wojkowska et al., 2014; Hou et al., 2019; Bao et al., 2023). Indeed, as early as 20 years ago, research indicated that treating adoptive EAE mice with anti-SR-PSOX/CXCL16 antibodies could reduce the incidence and nerve damage, and inhibit specific CD4^+^ cells, demonstrating that CXCR6/CXCL16 may be a potential target for the treatment of EAE (Fukumoto et al., 2004).

In addition to the high expression of peripheral and intracerebral CXCR6^+^CD4 T cells in the EAE mouse model, an increase in the number of CXCR6^+^CD8^+^ T cells has also been reported in the brains of MS patients (Fransen et al., 2020). During the disease course, 16.3% of CD8^+^ T cells reside in the brain parenchyma, and these cells exhibit a tissue-resident memory T cell phenotype with upregulated expression of CXCR6 markers (Fransen et al., 2020). In line with this, CXCL16 is highly expressed in all myeloid cells, and its specific receptor, CXCR6, is highly expressed in all CD8^+^ T cells. Subsequent research has shown that CXCR6^+^CD8^+^ T cells are essential for glial activation and synaptic pruning. These findings indicated that CXCL16/CXCR6 serves as a key signaling bridge in the interaction between T cells and microglia in the CNS (Rosen et al., 2022). In addition, spinal cord injury activates microglia and macrophages to produce the chemokine CXCL16, which recruits CXCR6^+^CD8^+^ T cells, resulting in the serious consequence of neuronal loss (Ge et al., 2025). Consequently, regulation *via* the CXCR6/CXCL16 axis may be essential for the pathogenic CXCR6^+^CD8^+^ T cells involved in neuroinflammation in MS.

Rutin, a natural flavonoid, is widely found in various botanical medicines, vegetables, and fruits commonly encountered in daily life and has rich pharmacological activities (Cui et al., 2026). Rutin has been reported by Ge et al. (2025) to promote motor recovery following spinal cord injury through targeting the CXCR6/CXCL16 axis. This study suggests that rutin may have potential neuroprotective and myelin-repairing properties. However, since MS models are primarily based on an autoimmune pathogenesis, further validation is needed to determine whether this metabolite also exerts its effects *via* the CXCR6/CXCL16 axis in MS disease models. Ying et al. (2023) reported that baicalein intervention in MOG_35–55_-induced EAE mice significantly reduced the expression of CXCR6^+^CD4 T cells and CXCR6^+^CD8 T cells, as well as the levels of CXCR6^+^IL-17A and CXCR6^+^IFN-γ. These results suggest that the mechanism by which baicalein alleviates clinical symptoms of EAE may involve the suppression of CXCR6-expressing pathogenic Th cells. Although the CXCR6/CXCL16 axis has been implicated in MS-related neuroinflammation (Bao et al., 2023), the current evidence supporting its modulation by TCM or TCM-derived metabolites remains very limited. The available data are primarily limited to baicalin and rutin, and since the mechanism of action of rutin has not been studied in the EAE model, the evidence is insufficient. Therefore, the CXCR6/CXCL16 axis should be regarded as a mechanism that gives rise to hypotheses, and further research is still needed to obtain more conclusive evidence.

**FIGURE 1:**
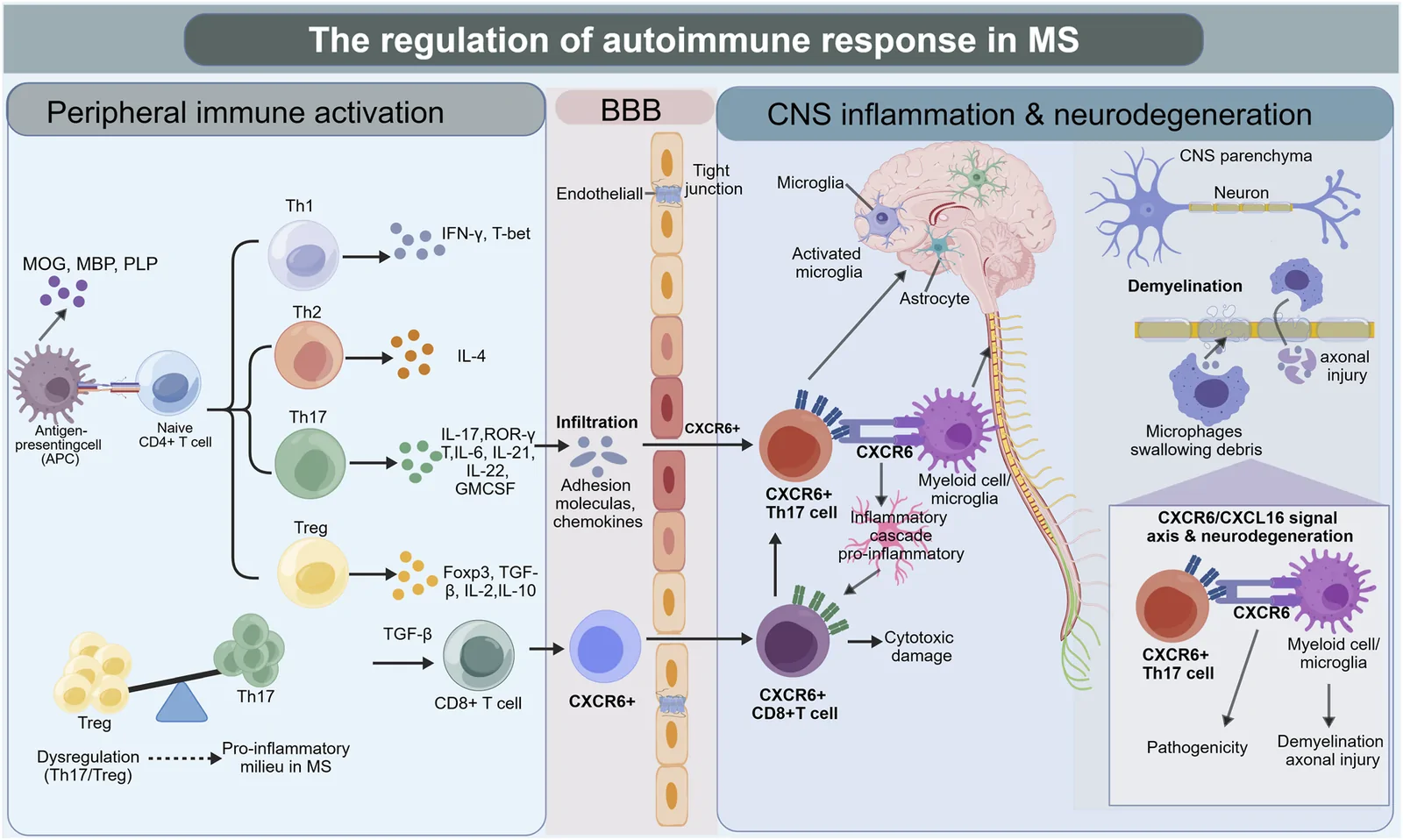
The regulation of autoimmune response in MS. Antigens (MOG, MBP, PLP) stimulate CD4^+^ T cells, primarily eliciting responses from T helper cells, including Th1, Th2, Th17, and Treg cells. Among these, Th1 and Th17 cells are pathogenic, while Th1 and Treg cells release anti-inflammatory factors that, in turn, suppress excessive Th17 cell activation. An imbalance between Th17 and Treg cells increases the severity of MS. Peripheral immune dysregulation disrupts the permeability of the BBB, allowing immune cells to infiltrate the CNS, where they promote abnormal activation of neurons, further contributing to demyelination and axonal damage in MS. In addition, CXCR6^+^CD4^+^ T cells and CXCR6^+^CD8^+^ T cells are highly expressed in the progression of MS, and the regulation of the CXCR6/CXCL16 signaling axis plays a crucial role. Created with BioGDP.com.

In conclusion, the dynamic balance between pro-inflammatory and anti-inflammatory factors influences the progression of MS. This section primarily summarizes and analyzes how TCM exerts its anti-MS effects by modulating the body’s immune response, as well as the underlying mechanisms (Figure 1; Table 2).

**TABLE 2:** The anti-MS effect of TCM in regulating the autoimmune system.

Type	Name	In vitro	In vivo	Dosage and duration	Treatment	Sample	Major findings	Limitation	References
Extract mixture	Astragalus polysaccharide	N/A	MOG_35–55_ -induced female C57BL/6 mice	500 mg/kg/d i.g. ⨯19 days	Prophylactic	Brain/colon/feces/plasma	↓IL-17A, IL-22 ↑IL-4, IL-10, TGF-β ↓Th17 cells ↑Treg cells ↓S1P, PGE2, ADP, and ATP	Single dose only; no positive control	(Ma et al., 2025)
Active metabolites	Ginkgolide K	N/A	MOG_35–55_ -induced female C57BL/6 mice	7.5 mg/kg i.p. ⨯19 days	Prophylactic	Spleen/spinal cord	↓Th17 cells ↑Treg cells ↑Macrophage/microglia M2 polarization ↓CCL-2, CCL-3, CCL-5 ↓TLR4, NF-κB, COX2	Single dose only; no positive control; no PK data; precise targets remain unclear	(Yu et al., 2019)
	β-elemene	Lymphocytes	MOG_35–55_ -induced female C57BL/6 mice	20 mg/kg i.p. ⨯19 days	Prophylactic	Spinal cord/lymph node	↓IL-17, IL-6, IL-23, RORγt ↓Th17 cells ↑Treg cells	Single dose only in vivo; no positive control; no PK data; mechanism primarily correlative	(Zhang et al., 2011)
	Baicalin	CD4^+^ T cell differentiation	MOG_35–55_ -induced female C57BL/6 mice	100 mg/kg/d i.p. ⨯20 days	Prophylactic and therapeutic (day 10/15 p.i.)	Spleen/lymph nodes/spinal cord	↓IL-17A, IFN-γ, GM-CSF, IL-1β, IL-6, IL-1, IL-23, TGF-β ↓MMP3 ↓CXCL-9, CXCL-10, CXCL11, CXCL1, CXCL2, CCL20 ↓T-bet, ROR-γt	No positive control; poor oral bioavailability; single dose only in vivo	(Zhang et al., 2015)
	Kurarinone	CD4_+_ T cell differentiation	MOG_35–55_ -induced female C57BL/6 mice	100 mg/kg/d i.p. ⨯20 days	Prophylactic and therapeutic (day 10 p.i.)	Brain/spleen/spinal cord	↓CD4+ and CD8^+^ T-cell ↓Th1 cells ↓Th17 cells	Single dose only in vivo; no positive control; no PK data; mechanism primarily correlative	(Xie et al., 2018)
​	Eriocalyxin B	CD4+ T cell differentiation	MOG_35–55_ -induced female C57BL/6 mice or splenocytes	10 mg/kg/d i.p. ⨯17 or 30 days	Prophylactic and therapeutic (day 11 p.i.)	Spleen/spinal cord	↓CD4+T cells, CD11b+, CD4^+^/IFN-γ^+^, CD4^+^/IL17^+^ cells ↓IFN-γ, IL17, T-bet, ROR-γt ↓p-STAT1, p-STAT4, p-STAT3, p-Jak1/2 ↓Th1 cells ↓Th17 cells	Single dose only in vivo; no PK data; no oral bioavailability data	(Lu et al., 2013)
	Daphnetin	LPS-induced BMDCs and RAW264.7 cells	MOG_35–55_ -induced female C57BL/6 mice	8 mg/kg i.p. ⨯28 days	Prophylactic	Spleen/spinal cord	↓Th1 cells ↓Th17 cells ↓NF-κB	Single dose only in vivo; no positive control; no PK data	(Wang et al., 2016)
	Berberine	Naive CD4+ T cells purified via MACS cell differentiation	MOG_35–55_ -induced male C57BL/6 mice	200 mg/kg i.g. ⨯9 days	Prophylactic and therapeutic (day 9 p.i.)	Spleen/spinal cord	↓Th1 cells ↓Th17 cells ↓CD11b+ APCs ↓NF-κB	Single dose only in vivo; no positive control; no PK data	(Qin et al., 2010)
	Anemoside A3	PGE2 or 15-OH PGE1-induced THP-1 cells	MOG_35–55_ -induced C57BL/6 mice	100 mg/kg, 10 mL/kg i.g. ⨯12 or 20 days	Prophylactic and therapeutic (day 8 p.i.)	Spleen/spinal cord	↓IFN-γ, IL-4, IL17 ↓p-STAT4, p-STAT3 ↑p-STAT6 ↓Th1 cells ↓Th17 cells	Single dose only in vivo; no positive control; no PK data; no minimal effective concentration	(Ip et al., 2017)
	Paeoniflorin	LPS-induced BMDCs	MOG_35–55_ -induced male C57BL/6 mice	5 mg/kg/d i.p. ⨯19 days	Prophylactic	Spleen/spinal cord	↓CD4+ and CD8^+^ T cells, CD4+IL-17+ cells ↓IL-17, IL-6, CD80, CD40, MHC II ↓pho-Ikkα/β, pho-IκBα and pho-NF-κB p65 ↓p-STAT3	Single dose only in vivo; no positive control; no PK data	(Zhang et al., 2017)
	Kirenol	MOG35-55-induced DLN lymphocytes	MOG_35–55_ -induced female C57BL/6 mice	2 mg/kg i.g. ⨯25 days	Prophylactic	Serum/lymph nodes	↓IFN-γ, IL-17A ↓Th1 cells ↓Th17 cells ↑Bax, caspase-3 ↓Bcl-2	Single dose only in vivo; no positive control; no PK data	(Xiao et al., 2015)
​	Plumbagin	LPS-induced DC2.4 cells	MOG_35–55_ -induced female C57BL/6	3 or 5 μM ⨯33 days	Prophylactic	Spleen/spinal cord	↓IL-4, GM-CSF ↓CD83, CD86, CD80, and HLA-DR	Single dose only; no positive control; no PK data	(Zhang et al., 2014)
	Arctigenin	CD4+ T cell differentiation; PMA- Stimulated Jurkat cells	MOG_35–55_ -induced female C57BL/6 mice	5 or 10 mg/kg i.p. ⨯28 or 18 days	Prophylactic	Spleen/spinal cord/lymph node	↓IFN-γ, T-bet, IL-17A, IL-17F, ROR-γt ↓p-P38 ↑PPAR-γ ↓Th1 cells ↓Th17 cells	No positive control; no PK data	(Li et al., 2016)
	Ginsenoside Rd	MOG35-55-induced Splenocytes	MOG_35–55_ -induced C57BL/6 mice	40 mg/kg/d i.g. ⨯25 days	Therapeutic	Brain/spleen/spinal cord	↓IFN-γ, IL-4 ↓BDNF, NGF ↑Th2 cells	No positive control	(Zhu et al., 2014)
	Yakuchinone A	DLN cells	MOG_35–55_-induced female C57BL/6 mice	50 mg/kg i.p. ⨯15 days	Therapeutic (day 12 p.i.)	Brain/spleen/spinal cord	↓IL-17	Single dose only in vivo; no positive control	(Lin et al., 2020)
	Rutin	HEK293T cells transfected with CXCL16/CXCR6 plasmids	C57BL/6 mice or CXCR6-/C57BL/6J mice induced SCI and MCAO model	100 mg/kg i.g. ⨯35 days	Therapeutic	Spinal cord/brain/spleen	↓CXCL16 ↓CXCR6 ↓CD8+ T cells	Single dose only; no positive control; not directly translatable to MS/EAE without validation	(Ge et al., 2025)
	Baicalein	CD4+ T cell differentiation	MOG_35–55_ -induced female C57BL/6 mice	25 mg/kg i.g. ×21 days	Prophylactic	Spleen/spinal cord	↓IL-17A, IgG, IgG3 ↓CXCR6+ CD4 and CD8 cells ↓CXCR6+ Th17 cells	Single dose only; no PK data	(Ying et al., 2023)
Chinese botanical drugs	Zingiber officinale Roscoe	LPS-inducted BV2 cells SH-SY5Y cells	PLP139–151 and MOG_35–55_ -induced SJL female mice/CB57BL/6 female mice	200 mg/kg i.g. ×14 days	Therapeutic (day 14 p.i.)	Plasma/Brain/spinal cord	↓IL-6, IL-17, TNF-α	Single dose only; no PK data	(Borgonetti et al., 2025)
	Rhodiola rosea	N/A	MOG_35–55_-induced female C57BL/6 mice	50 mg/kg/d, 100 mg/kg/d, 200 mg/kg/d i.g. ×9 days	Therapeutic (day 12 p.i.)	Serum/spleen/spinal cord	↓IL-6, sIL-6R, IFN-γ, IL-17A ↑IL-4 ↓Th1 cells ↓Th17 cells ↑Treg cells ↓JAK1, JAK2, STAT3, ROR-γt	No PK data	(Lin et al., 2020)
	Alpinia oxyphylla Fruit	PMA and Ionomycin -stimulated EL4 cells with	MOG_35–55_-induced female C57BL/6 mice	300 and 1000 mg/kg i.g. ×14 days	Prophylactic and therapeutic (day 7 p.i.)	Brain/spleen/spinal cord/lymph nodes/brain	↓CD4^+^ T cells, CD8^+^ T cells, CD11b^+^ ↓IFN-γ, IL-17, T-bet, ROR*γ*t, IL-2, TNF-α	No PK data	(Huang et al., 2019)
Formulas	Sheng Jiang powder	N/A	MOG_35–55_-induced female C57/BL6 mice	6.165 g/kg and 12.33 g/kg i.g. ×20 days	Prophylactic	Serum/spleen/brain/spinal cord	↓IL-17A, TGF-β1, IgG ↑IL-10, Foxp3 ↓iNOS+ and Arg-1+ cells ↓5-LOX ↑12/15-LOX	No PK data; mechanism on lipid metabolism remains unclear	(Wu et al., 2025)
	Huangqi-Guizhi-Wuwu decoction	N/A	MOG_35–55_-induced female C57/BL6 mice	3.5, 7, 14 g/kg i.g. ×20 days	Therapeutic (day 7 p.i.)	Serum/spleen/brain/spinal cord	↓CD4^+^T cells ↑IL-10, Foxp3 ↓IFN-γ, IL-17, ROR-γT, T-bet ↓p-JAK1, p-JAK2, p-STAT1, p-STAT3, p-STAT4 ↑p-STAT5	No PK data; mechanism on lipid metabolism remains unclear	(Xu et al., 2024)
​	Pien Tze Huang	N/A	PLP139−151-induced female SJL/J mice	0.234 g/kg/d i.g. ×60 days	Therapeutic (day 10 p.i.)	Serum/spleen/spinal cord/brain	↓IFN-γ, IL-17A, ROR-γt, T-bet ↓Th1 cells ↓Th17 cells ↓p-STAT1, p-STAT4, p-STAT3, p-P65/P65	single dose only; no dose-response relationship	(Qiu et al., 2018)
	Bu Shen Yi Sui capsule	N/A	MOG_35–55_ -induced female C57BL/6 mice	3.02 g/kg i.g. ×40 days	Not reported	Spleen/brain/spinal cord	↓CD4 + IL-17+/CD4 + CD25 + FoxP3+ ratio ↓IL-17A, IL-6, IL-23, TGF-β1 ↑Foxp3	No PK data; formula composition complex with multi-target effects	(Zheng et al., 2015)

## 4 The effects of TCM on inflammatory signals in MS pathological progression

### 4.1 NLRP3 signal pathway

Inflammasomes have been extensively studied as key players in immune responses, and they are known to bridge innate and adaptive immunity (Zhang et al., 2023). The Nod-like receptor protein 3 (NLRP3) is one of the most extensively studied and significant inflammasomes. It consists of NLRP3, the adaptor protein apoptosis-related speck-like protein (ASC), and the effector protein procaspase-1 (Kaneko et al., 2024). The secretion of the pro-inflammatory cytokines IL-1β and IL-18 is mediated by mature caspase-1, which in turn triggers a series of immune or inflammatory responses (Saresella et al., 2023; Yin et al., 2025). Studies have found that K^+^ efflux (Xu et al., 2025), Ca^2+^ signaling (Dai et al., 2022), reactive oxygen species (ROS) (Zhou M.-Y. et al., 2026) and lipopolysaccharide (LPS) (Lian et al., 2025; Xu et al., 2026) can activate the NLRP3 inflammasome, further increasing the risk and severity of disease. The activation of NLRP3 inflammasomes promotes inflammatory progression in MS disease, further disrupting the BBB and causing neurological damage (Fetisova et al., 2017). Recent studies have confirmed that treating EAE mice with the NLRP3 inflammasome-selective inhibitor MCC950 reduced neuronal injury, demyelination and oligodendrocyte loss in their brains. This may be due to immunomodulation, which involves suppressing microglial activation and inflammatory responses (Coll et al., 2015; Hou et al., 2024). These studies indicate that suppressing NLRP3 expression can delay the progression of MS, thereby achieving therapeutic effects.

Forsythoside B (FB) is a phenylethanoid glycoside isolated from *Forsythia suspensa* (Thunb.) Vahl, rich in biological activities (Wang Y.-T. et al., 2023). Wang Y. et al. (2025) found that FB significantly alleviated clinical symptoms of EAE mice and reduced its incidence. During the progression of EAE, microglia and astrocytes are abnormally activated, and the NLRP3 inflammasome is activated, promoting the GSDMD-mediated pyroptosis responses. FB reverses this trend, thereby inhibiting neuroinflammatory damage. The absolute oral bioavailability of FB is relatively low, so it may be necessary to add a substance such as chito-oligosaccharide to promote the absorption of phenethyl glycoside (Zhou et al., 2014). Icariin, a flavonoid, is the core active metabolite derived from the botanical drug *Epimedium brevicornu Maxim.* has been shown to have neuroprotective properties in neurological disorders (Sharma et al., 2025). Gao et al. (2023) demonstrated that treatment of EAE mice with Icariin suppressed microglia cell activation, downregulated neuroinflammatory cytokines including IL-1β, IL-6, and TNF-α, and further inhibited the activation of the NLRP3 inflammasome to improve demyelination and neuronal loss. A human pharmacokinetic study demonstrated that the plasma concentration of icariin was extremely low after oral administration. It is metabolized in the intestines into icaritin, which then enters the bloodstream and exerts its effects (Teo et al., 2019). Therefore, icaritin is the primary active metabolite absorbed by the body. Although icariin binds well to the NLRP3 complex *in vitro* (Gao et al., 2023), its therapeutic effect on MS remains to be further investigated. Sinomenine is an alkaloid derived from the roots and stems of *Sinomenium acutum* (Thunb.) Rehder and E.H.Wilson. In a study by Kiasalari et al. (2021), it was revealed that injection of sinomenine alleviates the severity of MOG_35-55_-induced EAE mice, likely by reducing levels of IL-1β, IL-6, IL-18, TNFα, and IL-17A while increasing IL-10 levels, and by decreasing the expression of the inflammasome complex NLRP3, ASC, and caspase-1. The latest research indicates that miRNA-223-3p exosomes derived from sinomenine-treated microglia can suppress NLRP3-mediated neuronal pyroptosis, thereby exerting neuroprotective effects (Yang Q. et al., 2025). Wei et al. (2021) revealed that arctigenin exerts a neuroprotective effect by inhibiting neuronal hyperactivity and increasing calcium influx in EAE mice. Prolonged calcium influx synergistically activates the NLRP3 inflammasome with ATP, jointly regulating inflammatory immune responses (Hao et al., 2017). Research suggests that arctigenin may act indirectly on the NLRP3 inflammasome rather than targeting it directly.

### 4.2 TLR4/MyD88/NF-κB signal pathway

The TLR4/MyD88/NF-κB signaling cascade plays a pivotal role in the mechanisms underlying the prevention and treatment of inflammatory, immune, and neurological diseases (Wang et al., 2021). Toll-like receptors (TLRs) were first identified in fungal infection assays in *Drosophila* (Weiss and O’Neill, 2022). Among them, TLR4 is the only receptor capable of activating both the MyD88-dependent pathway and the interferon-beta (TRIF)-dependent pathway (Fu et al., 2025). Research suggests that TLR4, a key pattern recognition receptor, triggers downstream signaling *via* the MyD88 adaptor upon activation, ultimately activating the classic inflammatory regulatory pathway NF-κB (Park et al., 2025). It is well known that inhibitors of NF-κB (IκB) proteins typically bind to NF-κB family members in the cytoplasm, thereby increasing their phosphorylation levels. Upon stimulation, IκB kinases (IKKs) are activated, promoting the phosphorylation and degradation of IκB proteins, enabling inactive NF-κB to enter the nucleus with transcriptional activity (Chen and Greene, 2004). Moreover, the transcription factor NF-κB is activated only after translocation into the nucleus, a process stimulated by TNF-α, oxidative stress, and Ca^2+^, thereby exerting a pro-inflammatory effect (El Bekay et al., 2003; de Oliveira-Marques et al., 2007). NF-κB translocates into the nucleus, where it regulates the expression of a series of inflammatory mediators, including ROS, IL-1β, IL-2, TNF-α, and CD86, and promotes M1 polarization of microglia, leading to the production of abundant proinflammatory mediators (Yin et al., 2022; Huang et al., 2026). These processes induce severe inflammatory responses, leading to demyelination and neuronal damage in the central nervous system. TLR4, MyD88 and NF-κB are key mediators of inflammation, and inhibition of their activity promotes the transformation of microglia and astrocytes into anti-inflammatory phenotypes (Guo M.-F. et al., 2020). In a word, TLR4/MyD88/NF-κB signaling activates the transcription of proinflammatory mediators, promotes the development of a series of inflammatory responses, exacerbates neuroinflammation, and worsens the progression of MS (Jingjing et al., 2024).

Emodin is an anthraquinone derived from *Rheum palmatum* L. or *Rheum tanguticum* (Maxim. ex Regel) Balf. or *R. officinale* Baill., which possesses various bioactivities, including anti-inflammatory and anticancer properties. In EAE mice, emodin was found to inhibit the activation of the TLR4/MyD88 signaling pathway, reduce microglial inflammatory responses, and significantly improve clinical symptoms (Zheng et al., 2022). Although reports indicate that the drug has poor water solubility and bioavailability (Wang et al., 2026), this does not affect its candidate or therapeutic value (Zheng et al., 2022). However, while direct high-dose administration *via* gastric lavage has demonstrated efficacy against EAE, the choice of administration method for subsequent clinical translation warrants careful consideration. Ginsenoside Re has been reported by Oh et al. (2024) to reduce the activation of microglia and macrophages, while suppressing the expression of proinflammatory cytokines IL-1β and IL-6, as well as chemokines MIP-1α, MCP-1, and RANTES *via* inhibiting the TLR4/MyD88/NF-κB signaling pathway, thereby preventing disruption of the BBB and ultimately alleviating spinal cord injury in EAE mice. Celastrol, a quinonemethide triterpene, is the primary active metabolite in *Tripterygium wilfordii* Hook. f. Wang et al. (2015) reported that celastrol inhibits the nuclear transcription activity of NF-κB, thereby reducing central nervous system inflammation and myelin loss in EAE mice. Tripterygium glycosides, a mixture of total tripterygium lactone glycosides found in crude extracts, has been demonstrated by Qiu et al. (2017) to possess the ability to delay the progression and severity of EAE. This effect is achieved by inhibiting CNS inflammatory cell infiltration, promoting inflammatory cell apoptosis, and downregulating NF-κB and IL-2 expression. Ginkgolide K promotes the M2 polarization of macrophages or microglia while simultaneously reducing the expression levels of proinflammatory factors such as TLR4, NF-κB, and COX2 in the spinal cord of EAE mice (Yu et al., 2019). In the cuprizone (CPZ)-induced demyelinating model, Ginkgolide B promotes myelin synthesis by inhibiting the expression of NF-κB, iNOS, IL-1β, and TNF-α while upregulating Arg-1 and neurotrophic factors (Yin et al., 2019). Clinical studies have reported that Ginkgo biloba extract can alleviate fatigue and reduce the severity of symptoms in MS patients (Johnson et al., 2006), but it does not improve cognitive function (Lovera et al., 2012). Hence, the clinical efficacy of the isolated metabolites reported in Ginkgo requires further investigation. Daphnetin, a coumarin derivative isolated from *Daphne odora* Thunb., has been reported to significantly inhibit dendritic cell activation as well as suppress NF-κB signaling within cells, thereby reducing neuroinflammation and demyelination in EAE mice (Wang et al., 2016). Moreover, curcumin-loaded HPPS inhibited inflammatory damage to the BBB and reduced the incidence of EAE in mice by 70% *via* suppressing the activation of the NF-κB signaling pathway (Lu et al., 2020). A clinical study has further demonstrated the therapeutic efficacy of nanocurcumin in MS (Dolati et al., 2018). It modulates the NF-κB signaling pathway in the blood of MS patients, reducing the expression of inflammatory cytokines such as IL-1β, IL-6, and TNF-α. Curcumin, derived from the botanical drug *C. longa* L., is a lipophilic substance that exerts its therapeutic effects through a targeted delivery system in the form of nanoparticle formulations, thereby providing a development strategy for other extracts and metabolites with low bioavailability (Aiello et al., 2026).

Rhizomes of Panax japonicus (RPJ) have a long history of medicinal use in China. *In vivo* experimental studies have confirmed that RPJ possesses neuroprotective capabilities, effectively alleviating spinal cord injury in EAE mice and reducing peripheral blood levels of TNF-α, IL-6, and IL-1β. Evidence from BV-2 cell-based *in vitro* assays indicates that the anti-inflammatory effect of RPJ against LPS stimulation is attributed to blockade of the TLR4/MyD88/NF-κB signaling pathway (Wang J. et al., 2023). Radix Rehmanniae (RR) is a classic example of a botanical drug used to treat MS. Li et al. (2018) have published findings that RR suppresses the NF-κB signaling pathway in splenocytes from EAE mice, with significantly reduced expression of phosphorylated IKKα/β, IκBα, and p65 compared to the model group. However, clinical treatment regimens typically involve more than one medication.

### 4.3 MAPK signal pathway

Mitogen-activated protein kinases (MAPKs) constitute a highly conserved cascade system that transmits signals through a tertiary phosphorylation cascade (MAPKKKs→MAPKKs→MAPKs), thereby promoting the phosphorylation of downstream substrates and regulating the progression of diseases such as cancer, arthritis, autoimmune disorders, and the nervous system (Hashimoto et al., 2012). The classic MAPK pathway comprises extracellular signal-regulated kinase (ERK1/2), Jun N-terminal kinase (JNK), p38 kinase and ERK5, representing the most prevalent set of signal transduction cascades. The JNK and p38 MAPK signaling pathways can be activated by various cellular stresses such as oxidative stress, genotoxic stress, osmotic stress, and proinflammatory cytokines (TNF-α and IL-1β) (Kim and Choi, 2015). Activation of the p38 MAPK pathway promotes secondary neuroinflammation induced by spinal cord injury (Qian et al., 2020), and the activation of microglia and astrocytes is also inevitable (Liu et al., 2025). Further studies revealed that inhibiting the activation of p38-MAPK and ERK1/2 reduces CCL2/CCL5-induced T cell migration, thereby diminishing the damage caused by pathogenic T cells to the nervous system (Cheng et al., 2015). Interesting studies have found that CXCR7-mediated chemotactic activity of microglia is caused by ERK1/2 activation, while MAPK and JNK signals are not involved. Downregulation of p-ERK1/2 levels can effectively improve the clinical symptoms of EAE (Bao et al., 2016). Thus, the MAPK signaling cascade plays a crucial role in inflammatory responses and immune regulation.

Scutellarin, a flavonoid derived from *Scutellaria baicalensis* Georgi (Scutellariae Radix), exhibits neuroprotective effects. Zhao et al. (2025) revealed that scutellarin intervention can inhibit MAPK signaling activation, promote M2 polarization of microglia, and mitigate myelin cell damage. Zhang et al. (2025) demonstrated that ginsenoside Rb3 effectively alleviates CPZ-induced demyelination by inhibiting MAPK and JNK phosphorylation, thereby suppressing neuroinflammation. In fact, TNF receptor-associated factor 6 (TRAF6) is activated by ubiquitination, which in turn activates the downstream MAPK signaling pathway. While it is undeniable that ginsenoside Rb3 exerts its anti-inflammatory effects by inhibiting the MAPK pathway, TRAF6 may be the key target for treating MS because it binds more effectively. Mogroside-V is a saponin metabolite derived from the dried fruits of *Siraitia grosvenorii* (Swingle) C.Jeffrey ex A.M.Lu and Zhi Y.Zhang (Siraitiae Fructus), which was studied by Xie et al. (2025). Their research team discovered that it can inhibit pro-inflammatory polarization in macrophages or microglia, reducing the levels of IL-12, IL-6, IL-1β, and TNF-α they secrete, and it also suppresses the phosphorylation levels of P38, JNK, and ERK proteins, thereby alleviating symptoms of spinal cord injury. In a spinal cord injury model studied by Fu et al. (2023), formononetin was found to reduce inflammatory mediators TNF-α and IL-6 by inhibiting activation of the p38 MAPK signaling pathway, thereby improving neuromotor deficits and spinal cord injury. Anika et al. (2024) intervened in the MS model induced by ethidium bromide (EB) injection into the skull base (ICP) area with Embelin, which can reduce the levels of neuroinflammatory cytokines TNF-α, IL-1β, and IL-6, oxidative stress, neurotransmitter production, and alleviate neuropathic injury, and this process is closely associated with the regulation of the p38 MAPK signaling pathway. Although these studies used spinal cord injury and ethidium bromide-induced demyelination models, which differ from the autoimmune demyelination characteristic of EAE, their reported anti-inflammatory and myelin-protective effects are relevant to MS-related pathological processes and should therefore be interpreted as indirect, hypothesis-generating mechanistic evidence.

### 4.4 PI3K/Akt signal pathway

The phosphoinositide 3-kinase (PI3K)/protein kinase B (Akt) signaling pathway plays a crucial role in regulating diseases such as inflammation, autoimmune disorders, and cancer, and has been extensively studied (Patel and Mohan, 2005). Phosphatase and tensin homolog (PTEN) is a lipid phosphatase that negatively regulates PI3K signaling. The PI3K pathway is activated by upstream signals, where the p110 catalytic subunit promotes the conversion of phosphatidylinositol-4,5-diphosphate (PIP2) to phosphatidylinositol-3,4, 5-triphosphate (PIP3), while PTEN reverses this effect, interrupting signal transmission and exerting a negative regulatory effect on PI3K (Mukherjee et al., 2021). AKT is a member of the serine/threonine kinase family, comprising three domains: pleckstrin homology (PH), the kinase domain, and the regulatory carboxy-terminal linker (Johnson et al., 2006). AKT phosphorylation regulates multiple downstream signaling molecules, including but not limited to mammalian target of rapamycin complex 1 (mTORC1), glycogen synthase kinase-3 (GSK3), forkhead box class O family member proteins (FOXOs), and cAMP-response element binding protein (CREB), participating in the regulation of numerous biological processes (Hermida et al., 2017). Among them, mTOR is a key downstream target of the PI3K/Akt signaling pathway and is highly expressed in the blood of RRMS patients, particularly in females (Ashtiani et al., 2020). Another study found that mTORC1 plays an almost decisive role in myelin formation within the central nervous system, representing a dependency relationship, while mTORC2 exerts a lesser influence. Myelin-related lipogenesis and protein gene regulation also strongly depend on mTORC1 (Lebrun-Julien et al., 2014). Therefore, based on clinical data, effective natural medicines that target these pathways have become a hot topic in ethnopharmacology.

Gastrodin is a phenolic glycoside derived from the dried rhizomes of *Gastrodia elata* Blume, which exhibits pharmacological activities such as anti-inflammatory and antioxidant. The zebrafish model was used to demonstrate that gastrodin promotes myelin formation. In EAE mice, gastrodin was further confirmed to effectively enhance myelin regeneration, a process regulated by elevated phosphorylation levels of PI3K, AKT, and mTOR (Shi et al., 2025). Besides its immunomodulatory effects, APS can also suppress PI3K/Akt signaling pathway-mediated IL-1β or LPS-induced cellular inflammatory damage, enhance the function of the cellular endothelium barrier, and exert neuroprotective effects (Zhao et al., 2024). This extract exerts neuroprotective effects in EAE mice through multiple pathways and the PI3K/Akt pathway is only one of them and does not serve as a key target. To date, there have been no studies on the pharmacokinetics of APS in humans or on its efficacy in treating MS, and challenges remain in translating this research into clinical practice. Unlike APS, astragaloside IV has been clinically proven to possess anti-inflammatory effects (Zhang et al., 2024). It promotes glucocorticoid receptor-mediated dephosphorylation of PI3K and Akt *in vitro*, thereby inhibiting microglial activation and reducing the production of downstream pro-inflammatory mediators, demonstrating potential for alleviating neuroinflammation in MS (Liu et al., 2016). Research indicates that this metabolite has a high molecular weight and low permeability and fat solubility, making it difficult for it to cross the BBB and enter the brain (Zhao et al., 2023), thus, an innovative approach to its pathway of action is needed. Dihydroartemisinin (DHA), a derivative of artemisinin, is also the primary active metabolite of artemether in the body and exhibits significant bioactivity. Most evidence indicates that DHA can act through three pathways: (1) reducing oxidative stress damage; (2) inhibiting neuroinflammation; (3) decreasing apoptosis to achieve the treatment of neurodegenerative diseases including MS (Gao et al., 2020; Arthur et al., 2023). These effects are mediated through the regulation of the PI3K/Akt signaling pathway. Matrine, a quinoline alkaloid present in *Sophora flavescens* Aiton, has been observed to modulate myelin protein and oligodendrocyte numbers, as well as p-Akt, p-mTOR, and p-PI3K protein levels, suggesting it alleviates EAE symptoms by activating the PI3K/Akt/mTOR pathway (Liu et al., 2017).

A formula known as Bu Shen Yi Sui formula is designed to treat diseases resulting from multifunctional decline in the nervous, skeletal, and immune systems caused by kidney deficiency. Research has found that it promotes axonal regeneration in EAE mice by upregulating the BDNF/TrkB and PI3K/Akt signaling pathways (Zheng et al., 2019). Furthermore, Tripterygium glycoside tablets have been observed that by modulating the PI3K/AKT pathway, it reduces the mRNA expression of the spinal cord inflammatory factors IL-17A, IFN-γ, TNF-α, and IL-6, as well as the transcription factors T-bet and ROR-γt, thereby alleviating neuroinflammation and demyelination in EAE mice (Wang H. et al., 2025). Although these formulations are already in clinical use, evidence-based medical research is still needed to gather further supporting evidence.

### 4.5 JAK/STAT signal pathway

The cytokine-activated Janus kinase (JAK)–signal transducer and activator of transcription (STAT) pathway is a crucial signaling pathway primarily involved in regulating immune responses, inflammation, cellular homeostasis, and disease pathogenesis (Shuai and Liu, 2003). This pathway consists of tyrosine kinase-related receptors, JAK proteins, and STAT proteins. Specifically, the JAK protein family, as intracellular non-receptor tyrosine kinases, comprises four members: JAK1, JAK2, JAK3, and tyrosine kinase 2 (TYK2) (Henry and Jorgensen, 2023). A variety of cytokines bind to JAK kinases to activate them, thereby leading to STAT phosphorylation (Roskoski, 2016). The STAT family comprises seven members: STAT1, STAT2, STAT3, STAT4, STAT5a, STAT5b, and STAT6. STATs can only exert their regulatory activity after being transcribed into the nucleus, and the extent of their accumulation within the nucleus correlates with their ability to bind to DNA (Reich, 2013). The complexity of its functional regulation is established based on the diverse active metabolites of JAK/STAT signals. In the MS/EAE model, the JAK/STAT signaling molecule is abnormally activated, likely due to the disease-induced immune response mobilizing a large number of cytokines such as IL-12, IL-6, IL-17, IL-21, IL-23, GM-CSF, and IFN-γ, leading to the loss of expression of negative regulatory factors (Benveniste et al., 2014). Inhibition of this pathway suppresses the differentiation of Th1 and Th17 cells, inactivates myeloid cells and reduces the expression of pro-inflammatory cytokines and chemokines (Liu et al., 2014). Consequently, the inhibition of the JAK/STAT pathway may be beneficial for the clinical treatment of MS.

Magnolol, the primary active metabolite of *Magnolia officinalis* Rehder and E.H.Wilson, has exhibited the capacity to selectively suppress p-STAT3 and p-STAT4 expression in CD4^+^ and CD8^+^ T cells from the spleen of EAE mice, while reducing Th17/Treg cells, for the treatment of MS (Chen et al., 2023). If the issues of low solubility and bioavailability of magnolol can be addressed, it would increase the potential for clinical development (Qu et al., 2023). Guggulsterone, the active metabolite extracted from *Commiphora wightii* (Arn.) Bhandari, is an effective anti-inflammatory agent and exists in two stereoisomers (E− and Z-). Oral pharmacokinetic evaluations have shown that, due to its pronounced first-pass effect, systemic exposure levels in humans are low (Chhonker et al., 2016). In an EB-induced rat demyelination model, it exerts neuroprotective effects by reducing STAT3 levels and inflammatory mediators (Kumar et al., 2021). This may suggest that the active metabolites produced by the liver during the metabolism of guggulsterone play a role and still hold potential as drug candidates. Total flavonoids of Astragus can modulate the JAK/STAT signaling pathway to promote Treg differentiation and suppress the Th17/Th1 cells ratio, thereby improving motor deficits in EAE, reducing inflammatory damage, and alleviating demyelination (Han et al., 2023). Given their limited clinical validation, such total extracts should be considered as potential adjunctive candidates rather than standalone therapies. Another key metabolite, Astragaloside IV, has also been reported to inhibit the balance of Th1 and Th17 cell differentiation regulated by the JAK/STAT signaling pathway (Yang et al., 2019). Cornel iridoid glycoside, the active mixture obtained from *Cornus officinalis* Siebold and Zucc., has neuroprotective effects. Accumulating evidence demonstrated that it has inhibitory effects on the phosphorylation activity of the JAK/STAT pathway and suppressed the activation levels of microglia entering brain regions, regardless of whether it was applied to the MBP_68-86_ peptide-induced EAE model in female Lewis rats or the MOG_35–55_ peptide-induced EAE model in female C57BL/6J mice (Yin et al., 2014; Qu et al., 2019). Both EAE models confirmed the neuroprotective effects of this metabolite, making it a promising candidate for future clinical trials aimed at reducing neuroinflammation in MS. Luteolin, a naturally occurring flavonoid, promotes IFN-β-induced activation of the JAK/STAT signaling pathway by enhancing phosphorylation of JAK1, Tyk2, and STAT1/2 (Tai et al., 2014). Additionally, it alleviates neuroinflammatory damage in MS through three coordinated mechanisms: (1) regulation of inflammatory cytokine levels; (2) modulation of mitochondrial function; (3) improvement of cellular redox status, which is intrinsically linked to JAK/STAT inhibition (Mahto et al., 2025). Recent research indicates that encapsulating luteolin in extracellular vesicles enhances its permeability across the BBB, allowing it to be delivered directly to the central nervous system, and may serve as a novel therapy for neuroinflammation (Zhou W. et al., 2026). Anemoside A3 (AA3), a natural triterpenoid glycoside isolated from the roots of *Pulsatilla chinensis* (Bunge) Regel, has been identified for its ability to inhibit the activation of STAT4 and STAT3, downregulate the expression of Th1 and Th17 cytokines, thereby modulating the progression of EAE (Ip et al., 2017). This study focuses more on revealing how AA3 drives a shift toward a Th2 anti-inflammatory response and restores the Th1/Th2 balance. Therefore, JAK/STAT signaling is closely associated with immune regulation, reflecting the complexity of disease progression; simultaneously, it is important to consider the synergistic effects of multiple pathways in treatment.

Cellular and animal experimental studies have confirmed that Huangqi-Guizhi-Wuwu decoction can suppress the expression of p-JAK1, p-JAK2, p-STAT1, p-STAT3, and p-STAT4 proteins in lymphoid tissues of EAE mice, while increasing the expression of p-STAT5 protein. This regulation of inflammatory pathways contributes to alleviating nerve damage in MS (Xu et al., 2024). In addition, HGWD can increase levels of myelin-specific proteins and promote myelin regeneration, thereby alleviating chemotherapy-induced neuropathy (Yang X. et al., 2025). MS also belongs to demyelinating diseases. In terms of treatment strategies, it is necessary to both suppress immune inflammation and promote myelin regeneration. Shengjiang Powder, a classic formula preparation, demonstrates good anti-inflammatory properties. Lyu et al. (2022) discovered that the formulation downregulates the expression of IL-6, JAK2, and STAT3, and has an inhibitory effect on the IL-6/JAK2/STAT3 signaling pathway in EAE mice. This mechanism enables the formulation to mitigate BBB damage, cerebral tissue inflammation, and demyelination in EAE mice. These classic formulas of TCM are composed of multiple combinations of botanical drugs that work synergistically. Their efficacy has been proven through long-term clinical practice, and they have addressed the shortcomings of the first-pass effect of a single metabolite and lack of bioavailability.

**FIGURE 2:**
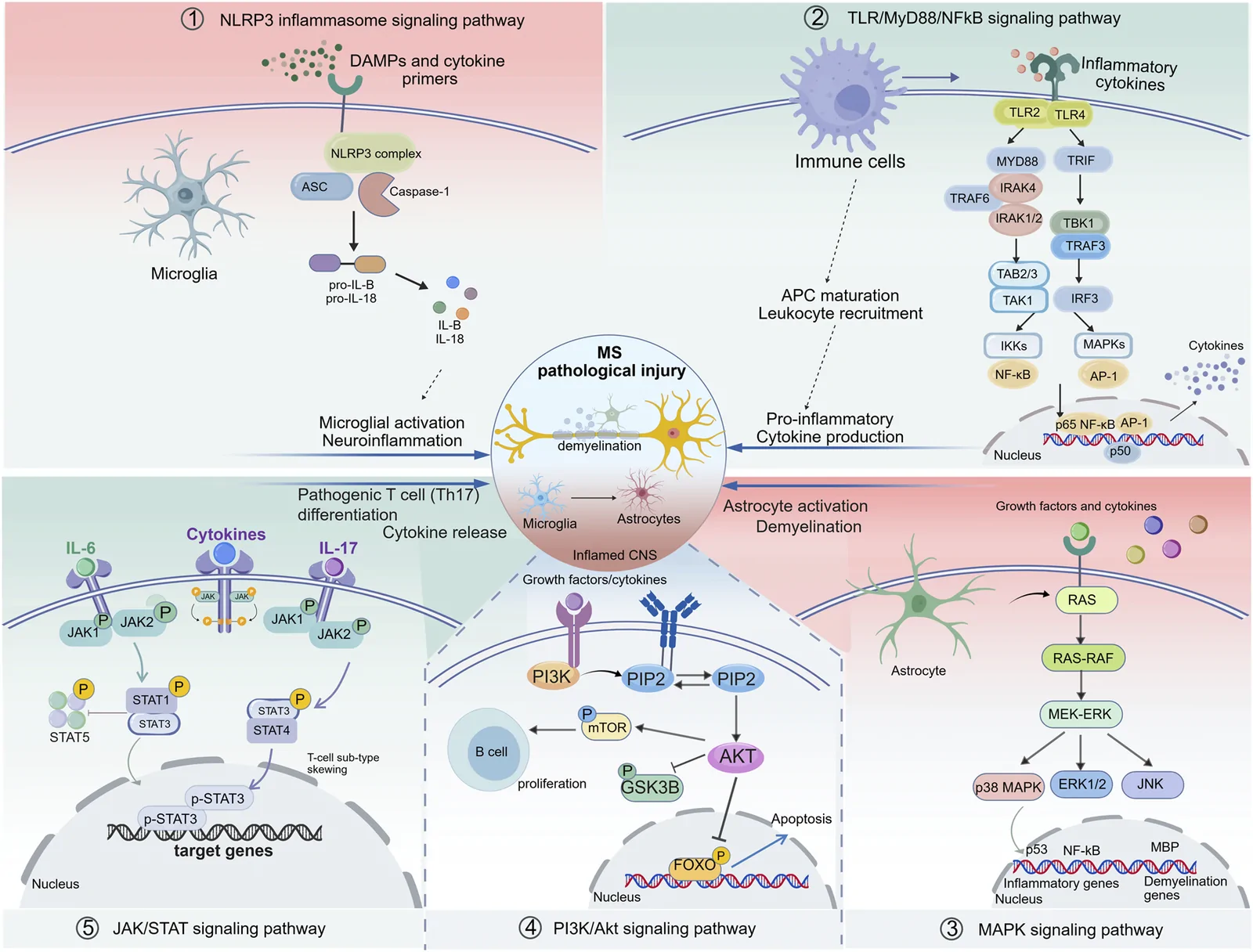
Regulatory interplay of key signaling pathways in MS. The regulation of inflammation-related signaling pathways contributes to the progression of MS, including the NLRP3 inflammasome pathway, TLR4/MyD88 signaling pathway, NF-κB signaling pathway, MAPK signaling pathway, PI3K/Akt signaling pathway, and JAK/STAT signaling pathway. They also interact with each other, activating macrophages, B cells, microglia, and astrocytes, which release pro-inflammatory factors that infiltrate the nervous system, thereby causing the pathological damage associated with MS. TCM exerts its therapeutic effects on MS by inhibiting the expression of these pathways. Created with BioGDP.com.

Based on the above studies, the regulation of inflammation-related signaling pathways in MS is shown in Figure 2. The isolated metabolites, botanical drugs and formulas of TCM demonstrate significant therapeutic effects on MS by interfering with inflammation-related signaling pathways, thereby revealing their underlying molecular mechanisms (Table 3).

**TABLE 3:** The anti-MS effect of TCM in regulating the inflammatory signaling pathway.

Type	Name	*In vitro*	*In vivo*	Dosage and duration	Treatment	Sample	Major findings	Limitation	References
Active metabolites	Forsythoside B	N/A	MOG_35–55_-induced male or female C57BL/6 mice	10 mg/kg or 40 mg/kg i.p. ⨯30 days	Prophylactic	Spinal cord	↓IL-6, TNF-α, IL-1β ↓Iba-1(microglia), GFAP (astrocytes) ↓NLRP3, caspase-1, c-caspase1, ASC, GSDMD ↑SOD, GSH	No positive control; no minimal effective concentration	(Wang et al., 2025b , Wang et al., 2023b)
	Icariin	N/A	MOG_35–55_-induced female C57BL/6 mice	7.5, 15 mg/kg/d i.p. ⨯28 days	Therapeutic (day 17 p.i.)	Brain/spinal cord	↓IL-1β, IL-6, TNF-α ↓NLRP3, caspase-1, c-caspase1, ASC	No minimal effective concentration no PK data	(Gao et al., 2023)
	Sinomenine	N/A	MOG_35–55_-induced C57BL/6 mice	25 or 100 mg/kg/d i.p. ⨯30 days	Therapeutic (day 11–12 p.i.)	Brain/spinal cord	↓IL-1β, IL-6, IL-18, TNFα, IL-17A ↑IL-10 ↓NLRP3, ASC, caspase 1 ↓Iba-1, GFAP	No positive control; no PK data	(Kiasalari et al., 2021)
	Arctigenin	N/A	MOG_35–55_-induced female C57BL/6 mice	10 mg/kg i.p	Prophylactic	Brain	↓AMPA- sEPSCs ↓Ca^2+^ influx ↓NLRP3, ASC, caspase 1	No positive control; no PK data; not directly act on NLRP3	(Hao et al., 2017)
	Celastrol	MOG_35–55_-induced Lymphocytes, Leukocytes, BMDCs, Naïve T Cells	MOG_35–55_-induced C57BL/6 mice	16 μg/200 μL/mice i.p. ⨯21 days	Prophylactic	Lymph node/spinal cord/spleen	↓IL-17, RORγt, STAT3 ↓IL-1β, IL-6, TNF-α ↓NF-κB, p-p65, IKK	No positive control; single dose only; no PK data; no minimal effective concentration	(Wang et al., 2015)
	Ginkgolide K	MOG-specific CD4 T cells	MOG_35–55_-induced female C57/B6 mice	7.5 mg/kg i.p. ⨯27 days	Pre-onset, from day 3	Spinal cord/spleen	↓TLR4, NF-κB, COX2 ↓CCL-2, CCL-3, CCL-5	No positive control; single dose only in vivo; no PK data	(Yu et al., 2019)
	Ginkgolide B	Astrocytes/LPS-induced BV2 cells/oligodendrocytes	CPZ-induced C57BL/6 male mice	20 mg/kg i.p. ⨯14 days	Therapeutic (week 4 p.i.)	Brain/spinal cord	↓TLR4, NF-κB, iNOS, IL-1β, TNF-α ↑Arg-1, BDNF	Single dose only in vivo; no positive control; no PK data	(Yin et al., 2019)
	Daphnetin	LPS-induced BMDCs and RAW 246.7 cells	MOG_35–55_-induced female C57BL/6 mice; BALB/c mice	8 mg/kg i.p. ⨯28 days	Prophylactic	Spleen/spinal cord	↓p-p65, p-IKK*α*/*β* ↑HO-1	No positive control; single dose only in vivo; no PK data NF-κ B studied only in vitro	(Wang et al., 2016)
	Curcumin	IFN-γ-activated b. End3 cells	MOG_35–55_-induced female C57BL/6 mice	1.8 mg/kg i.p. ⨯19 days	Prophylactic and therapeutic (day8, 10,12,14 p.i.)	Blood/brain/spinal cord	↓NF-κB, AP-1, IL-1β, IL-6, IFN-γ, CCL2, CCL5, TNF-α ↓ICAM-1, Mac-1	No positive control; single dose only	(Lu et al., 2020)
​	Emodin	N/A	MOG_35–55_-induced female C57BL/6 mice	30 mg/kg/d and 60 mg/kg/d ⨯21 days	Prophylactic	Brain/spinal cord	↓TLR4, MyD88, TICAM1, NF- κB ↓IL-6, TGF-β, IL-17A, RORγt	No positive control; single sample about mechanism; no PK data	(Zheng et al., 2022)
	Ginsenoside Re	LPS-stimulated bEND.3 cells	MOG_35–55_-induced female C57BL/6 mice	2.5, 5, and 10 mg/kg i.p ⨯30 days	Prophylactic	Brain/spinal cord	↓IL-1β, IL-6 ↓MIP-1α, MCP-1, RANTES, iNOS ↓TLR4/MyD88/NF-κB	No positive control; no PK data	(Oh et al., 2024)
	Scutellarin	Copper-induced BV2 cells and MO3.13 cells	N/A	20 µM ⨯24 days	Prophylactic	cells	↓p-p38 ↑p-ERK ↓MAG, MBP, TNF-α ↑TGF-β, Arg-1, Igf-1	No positive control; no PK data; single dose only; in vitro only	(Zhao et al., 2025)
	Ginsenoside Rb3	LPS- induced BV-2 cells; primary astrocyte; IL-1β-stimulated HeLa cells	CPZ- induced female C57BL/6 mice	20, 40, and 80 mg/kg i.g. ⨯4 weeks	Therapeutic (week 2 p.i.)	Brain	↓TNF-α, IL-6, IL-1β ↓p-NF-κB p65, p-MAPK p38, p-JNK ↓TRAF6 K63 ubiquitination	No PK data	(Zhang et al., 2025)
	Mogroside-V	BV2 cells	Female C57BL/6 mice SCI model	2.5 mg/kg, 5 mg/kg i.p. ⨯28 days	Therapeutic	Hearts/livers/spinal cord/supernatants	↓CD86 ↑CD206 ↓IL-6, IL-12, IL-1β, TNF-α ↓p-P65, p-p38, p-ERK, p-JNK	No positive control; no PK data; not directly translatable to MS/EAE without validation	(Xie et al., 2025)
	Formononetin	IL-1β- stimulated primary microglia/HMC3 cells	Rats SCI model	i.p	Therapeutic	Spinal cord	↓TNF-α, IL-6 ↓p-EGFR, p-p38	No positive control; no PK data; not directly translatable to MS/EAE without validation	(Fu et al., 2023)
	Embelin	N/A	EB- Wistar rats	1.25, 2.5, 5 mg/kg ⨯28 days	Therapeutic	Serum/spinal cord/brain	↓TNF-α, IL-1β, IL-6 ↓AchE, SOD, MDA, GSH, nitrite ↑GABA, DA, 5-HT	No positive control; no PK data; not directly translatable to MS/EAE without validation	(Anika et al., 2024)
	Gastrodin	OPCs or MO3.13 cells	LPC or MOG_35–55_-inducedfemale C57BL/6 mice or SD rats or zebrafish	100 mg/kg/d i.p. ⨯30 days	Not reported	Brains/spinal cords/serum	↑p-PI3K, PI3K, p-AKT, AKT, p-mTOR, mTOR, MBP	Single dose only; no PK data; lack of counterexample experiments	(Shi et al., 2025)
	Dihydroartemisinin	N/A	LPS-induced male and female C57BL6 mice	40 mg/kg i.p. ⨯19 days	Therapeutic	Brain	↓IL-1β, IL-6 ↓caspase-3, cleaved caspase-3 ↓P-PI3K/PI3K, P-AKT/AKT	Single dose only; no positive control; no PK data; not directly translatable to MS/EAE without validation	(Gao et al., 2020)
​	Matrine	N/A	MOG_35–55_-induced female C57BL/6 mice	200 mg/kg/d i.p. ⨯10 days	Therapeutic (day 13 p.i.)	Spinal cord/brain	↓CC1+ cells ↑p-PI3K, p-Akt, p-mTOR, p-P70S6K	Single dose only; no PK data	(Liu et al., 2017)
	Astragaloside IV	LPS-induced BV2 cells	MOG_35–55_-induced female C57BL/6 mice	20 mg/kg/d i.p. ⨯3 weeks	Therapeutic	Spinal cord/cells	↓IL1β, TNFα, iNOS, CD11b ↓p-PI3K, p-Akt ↓p-NF-κB, p-IκB	Single dose only; no PK data	(Liu et al., 2016)
	Magnolol	Th17 and Treg cell differentiation	MOG_35–55_-induced male C57B/L6 mic	10 mg/kg/d and 30 mg/kg/d i.p. ⨯12 days	Therapeutic (day 10 p.i.)	Spleen/serum/spinal cord	↓IL- 6, IL-17A ↑Th17/Treg cells ↓p-JAK2, p-STAT3	Non-immune cell line; no PK data; male mice only	(Chen et al., 2023)
	Guggulsterone	N/A	EB-induced Wistar rats	30 mg/kg and 60 mg/kg p.o. ⨯35 days	Therapeutic	Brain/serum	↓STAT-3 ↑PPAR-γ, MBP ↓TNF, IL-1β, AchE SOD, catalase, MDA, GSH, nitrite ↓caspase-3, Bax ↑Bcl-2	No positive control; no PK data; not directly translatable to MS/EAE without validation	(Kumar et al., 2021)
	Astragaloside IV	PBMCs or MNC or Naïve CD4 T cells	MOG_35–55_-induced female C57BL/6 or PLP139–15- induced SJL mice	20 mg/kg i.p. ⨯22 days	Therapeutic (day 9 p.i.)	Spinal cord/blood/brain/spleen/lymph nodes	↓RORγt, T-bet ↑Foxp3, Bcl-2 ↓p-JAK1, p-JAK2, p-STAT1, p-STAT3, p-STAT4 ↓p-NF-κB, p-IκB, bax	Single dose only; no PK data; no positive control	(Yang et al., 2019)
	Anemoside A3	THP-1 cells	MOG_35–55_-induced C57BL/6 mice	100 mg/kg,i.g ⨯20 days	Prophylactic and therapeutic (day 8 p.i.)	Spleen/spinal cord	↓IFN-γ, IL-4, IL17 ↓p-STAT4, p-STAT3 ↑p-STAT6 ↓Th1 cells ↓Th17 cells	No PK data; no positive control; single dose only	(Ip et al., 2017)
Extract mixture	Tripterygium glycosides	N/A	MBP-induced male Lewis rats	10 mg/kg/d i.g. ⨯21 days	Prophylactic	Blood/brain/spinal cord	↓IL-2 ↓NF-κB p65	No positive control; single dose only; no PK data	(Qiu et al., 2017)
	Astragalus polysaccharide	IL-1β or LPS-induced bEnd.3 cells	LPS-induced female C57BL/6 mice	500 mg/kg/d i.g. ⨯26 days	Therapeutic (day 7 p.i.)	Brain/spinal cord	↓VCAM-1, CD8+T cells ↑ZO-1, VE-cadherin ↓p-PI3K, p-Akt	Single dose only; no PK data; not directly translatable to MS/EAE without validation	(Zhao et al., 2024)
	Total flavonoids of Astragus	Differentiation of CD4+ T cells	MOG_35–55_-induced female C57BL/6 mice	50 mg/kg i.p. ⨯13 days	Therapeutic (day 9 p.i.)	Spinal cord/blood/brain/lymph nodes	↑IFN-γ, IL-10 ↓IL-17 ↓p-JAK1, p-JAK2, p-STAT1, p-STAT3, p-STAT4 ↑Foxp3, p-STAT5 ↓p-NFκB, p-IκB	Single in vivo dose only; no PK data	(Han et al., 2023)
​	Cornel iridoid glycoside	N/A	MBP_68–86_-induced female Lewis rats	30, 60 or 120 mg/kg i.g. ⨯20 days	Prophylactic	Spinal cord	↓TNF-α, IL-1β, IFN-γ ↑BDNF ↓p-JAK1, p-JAK3, p-STAT1, p-STAT3, NF-κB	No PK data; no positive control; no minimal effective concentration	(Yin et al., 2014)
	Cornel iridoid glycoside	LPS/IFN-γ and IL-4/IL-10- stimulated BV2 cells; N9 cells	MOG_35–55_-induced female C57BL/6J mice	50 or 100 mg/kg i.g. ⨯24 days	Prophylactic	Brain	↓IL-6, NO, TNF-α, IFN-γ, IL-6, MCP-1, IL-12 (p35) and IL-12 (p40) ↓p-JAK1, p-JAK3 ↑SOCS1, IL-10	No PK data; no positive control; no primary microglia	(Qu et al., 2019)
Chinese botanical drugs	Radix Rehmanniae	LPS- induced SH-SY5Y cells or RAW264.7 cells	MOG_35–55_-induced female C57BL/6N mice	3.7 g/kg/d i.g. ⨯30 days	Prophylactic and therapeutic (day 11 p.i.)	Spleen/brain/spinal cord	↓p-IKKα/β, p-IκBα, p-p65 ↓ROS/RNS ↓iNOS, NAPDH	No PK data; no positive control; single dose only	(Li et al., 2018)
	Rhizomes of Panax japonicus	LPS-treated BV2 cells	MOG_35–55_-induced female C57BL/6 mice	36.5 mg/kg, 73 mg/kg i.p. ⨯28 days	Prophylactic	Serum/brain/spinal cord	↓IL-17a/Foxp3, Th17/Treg, Th1/Th2 cells ↓TNF-α, IL-6, IL-1β ↓TLR4, MyD88, NF-κB	No PK data; pathway studied only in vitro	(Wang et al., 2023a)
Formulas	Bu Shen Yi Sui Formula	SH-SY5Y cells	MOG_35–55_-induced male SD rats or female C57BL/6 mice	11.7 g/kg i.g. 3.02 g/kg ⨯40 days	Prophylactic	Brain/spinal cord/serum	↑BDNF, TrkB ↑p-PI3K/PI3K, p-ERK/ERK ↑GAP-43, p-CREB/CREB	No PK data; no BBB function evaluation	(Zheng et al., 2019)
	Tripterygium glycoside tablets	N/A	MOG_35–55_-induced female C57BL/6 mice	10 mg/kg/d and 20 mg/kg/d i.g. ⨯22 days	Therapeutic (day 10 p.i.)	Spinal cord/blood/	↓IL-17A, IFN-γ, TNF α, IL-6 ↓T-bet, ROR-γt ↓p-PI3K, p-AKT, p-NF-κBp65, BAX ↑BCL-2	No positive control; no PK data	(Wang et al., 2025a)
	Huangqi-Guizhi-Wuwu decoction	Th1/Th17/Treg polarization and differentiation	MOG_35–55_-induced female C57/BL6 mice	3.5, 7, 14 g/kg i.g. ⨯15 days	Therapeutic (day 7 p.i.)	Serum/spleen/brain/spinal cord	↓p-JAK1, p-JAK2, p-STAT1, p-STAT3, p-STAT4 ↑p-STAT5	No positive control; no PK data; remyelination not assessed	(Xu et al., 2024)
	Shengjiang Powder	N/A	MOG_35–55_-induced female C57 BL/6 mice	246.6, 493.2, 739.8 mg/kg i.g. ⨯21 days	Prophylactic	Brain	↑Occluding, claudin-5 ↓IL-6, p-JAK2, p-STAT3 ↑MBP	No PK data; no BBB function evaluation	(Lyu et al., 2022)

## 5 Conclusions and perspective

Based on existing understanding, it is believed that MS is a complex and refractory neuroinflammatory disease due to a myelin regeneration disorder. In this review, we conclude that single botanical drugs, active metabolites, or formulas play a significant role in the treatment of MS. They are capable of inhibiting the differentiation and migration of pathogenic T cells, restoring the Th17/Treg cells imbalance, and suppressing the CXCR6/CXCL16 signaling axis, thereby modulating neuroimmune responses. In addition, TCM modulates inflammation-related signaling pathways such as the NLRP3, TLR/MyD88/NF-κB, MAPK, PI3K/Akt, and JAK/STAT pathways. The same medicine exerts both immunomodulatory effects and inhibitory actions on inflammatory signal transduction, which intuitively reflects the multi-component, multi-target and multi-pathway characteristics of TCM. However, the numerous studies on the use of TCM for treating MS are not without limitations that warrant acknowledgment.

Despite the encouraging preclinical data, several key challenges must be addressed before metabolites can be translated into clinical applications. First, most studies have relied on *in vivo* and *in vitro* experiments to validate the efficacy of these treatments. In cell experiments can only provide hypotheses regarding mechanisms and they do not reflect clinical symptoms; for example, cytotoxicity does not reflect liver or kidney toxicity. In animal studies, the MOG_35-55_ peptide, MBP_68-86_, and CPZ are commonly used to establish EAE demyelination models; It is worth noting that these EAE animal models can only reproduce some of the pathophysiological features of MS and cannot fully simulate the full spectrum of the human disease. The anti-inflammatory and myelin-protective effects of some bioactive metabolites have been studied in spinal cord injury, ethidium bromide-induced demyelination, or LPS-induced neuroinflammation models. However, these findings cannot be considered direct evidence for MS and still need to be validated in EAE or other MS-related models in the future. Second, the pharmacokinetic profile of the isolated metabolites or extracts is not yet fully understood and requires further validation. Most molecules have low oral bioavailability and are subject to the first-pass effect, for example, guggulsterone. Advanced drug delivery strategies were used to enhance bioavailability and enable targeted delivery to inflamed tissues, such as nanocurcumin. Icariin is present at extremely low levels in human blood and it is metabolized in the gut into icaritin, which exerts its biological activity, so the regulatory effects are not entirely due to the parent metabolite. Third, in the current original research, both prophylactic and therapeutic approaches were widely adopted and relatively evenly distributed among the compounds examined for their effects on the disease. This balanced distribution suggests that the medicines reviewed here may hold translational promise for both disease prevention and treatment, which should be considered when evaluating their clinical potential.

At present, the clinical evidence supporting the use of TCM to treat MS remains mixed (Table 4). Some clinical studies suggest that TCM or certain prescriptions may offer potential benefits in alleviating MS symptoms and reducing relapse rates, such as Frankincense, the Shugan Jianpi Gusui recipe, and the Erhuangfang formula (Zhou et al., 2013; Zhou and Fan, 2015; Stürner et al., 2018). However, several clinical trials have yielded negative results. Although American ginseng water extracts can significantly reduce demyelination scores and the expression of iNOS and TNF-α in the spinal cords of EAE mice (Bowie et al., 2012), clinical trials have found that American ginseng does not alleviate fatigue in MS patients (Kim et al., 2011). Furthermore, trials of Epigallocatechin-gallate (EGCG) in patients with MS have not demonstrated consistent efficacy, have shown no neuroprotective effects, and may be associated with an elevated risk of hepatic and renal toxicity (Lovera et al., 2015). A recent Phase II randomized controlled clinical trial also confirmed that although oral lipoic acid has potential neuroprotective bioactivity, it failed to improve walking function in people with progressive MS (Spain et al., 2026). These negative results suggest that a significant translational gap still exists between preclinical models and human MS, and that the immunomodulatory or neuroprotective mechanisms observed in experimental models are difficult to directly translate into clinical benefits. There are many factors contributing to this discrepancy, which may include species differences, heterogeneity among MS subtypes, differences in disease stage, and insufficient dosing, among others. Therefore, in the absence of sufficient evidence from clinical trials, active metabolites of TCM are better regarded as potential adjunctive therapies or candidates for new mechanisms of action, rather than being described as established disease-modifying treatments for MS.

**TABLE 4:** Clinical evidence of TCM in MS.

Name	MS subtype	Dosage and duration	Sample size	Main outcome	Result	Interpretation	References
Frankincense	RRMS	4800 mg/d ×8–36 months	38	Contrast-enhancing lesions	Positive	↓TNF-β, IL-4, IL-5, GM-CSF, IL-17A, IL-2, and IFN-γ ↑Treg cells, IL-10	(Stürner et al., 2018)
Crocin capsules	MS	2 crocin/d ×28 days	40	Inflammatory markers/oxidative damage/DNA damage	Positive	↓TNF-α, IL-17 ↑antioxidant capacity	(Ghiasian et al., 2019)
Saffron syrup	MS	7.5 cc/8 h ×2 months	30	Fatigue	Positive	↓Fatigue severity	(Ashtiani et al., 2020)
Ginkgo biloba extract	MS	240 mg/d ×12 weeks	61	Cognitive function	Negative	No improve cognitive performance	(Lovera et al., 2012)
Nanocurcumin	RRMS	6 months	50	miRNAs/transcription factors/pro-inflammatory cytokines	Positive	↓miR-145/miR-132/miR-16/STAT1/NF-κB/AP-1/IL-1β/IL-6/IFN-γ/CCL2/CCL5/TNF-α	(Dolati et al., 2018)
Shugan Jianpi Gusui recipe	RMS	10–131 months	14	Recurrence intervals and the yearly average recurrence times	Positive	Prolongs the recurrence interval and reduces the yearly average recurrence times	(Zhou et al., 2013)
Erhuangfang	RMS	2-year follow-up	67	Relapse rate	Positive	Reduces relapse rate and prevents progression of MS	(Zhou and Fan, 2015)
American ginseng	MS	100 mg, 200 mg, 400 mg/d ⨯6 weeks	56	Fatigue	Negative	No improve fatigue	(Kim et al., 2011)
Epigallocatechin-gallate	MS	Phase I: 400 mg/d ⨯6 months Phase II: 400 mg/d ⨯12 months	10/12	Adverse events/plasma levels and change in N-acetyl aspartate (NAA)	Negative	No correlation with NAA changes or high risk of hepatotoxicity in some EGCG	(Lovera et al., 2015)
Lipoic acid	PMS	1200 mg/d oral ⨯24 months	118	Timed 25-Foot Walk/brain atrophy	Negative	No slow declines in walking speed or no statistically significant	(Spain et al., 2026)

TCM and most commonly found plant metabolites or mixtures offer advantages such as low toxicity, safety, and low cost. In addition, these substances are commonly found in a variety of sources, such as rutin. In terms of safety, both single and multiple intravenous infusions of ginkgolide K and ginkgolide B were well tolerated, and no significant synergistic toxicity was observed when administered in combination with midazolam (Shao et al., 2017). Of course, to gain a clear understanding of the safety data for these drugs, a comprehensive toxicological assessment including evaluations of genotoxicity, reproductive toxicity, and immune toxicity is essential to ensuring clinical safety. Moreover, TCM interventions may represent potential adjunctive candidates for MS, but their combined use with standard disease-modifying therapies requires further safety evaluation, including assessment of herb-drug interactions.

Future research should prioritize well-designed preclinical and clinical studies. Concerning therapeutic relevance, this requires supporting evidence from pharmacokinetic studies, including oral bioavailability, BBB penetration, and *in vivo* exposure, as well as data from adequately designed clinical trials. For potential targets identified in in vivo studies, such as CXCR6/CXCL16, further investigations using gene editing, targeted inhibition, and functional validation experiments should be conducted to clarify their causal role in MS/EAE-related neuroinflammation. By leveraging the diversity of plant resources, future research should focus on investigating the biological activity of more isolated metabolites and the permeability advantages of small molecules across the BBB. Of course, we still cannot ignore the classic TCM preparations that have been circulating for thousands of years. Their effectiveness has been verified by a large body of clinical practice, but more intuitive evidence-based medicine is needed to convince more people.

In summary, current evidence suggests that various TCM preparations and active metabolites exhibit promising immunomodulatory and neuroprotective effects in preclinical models of MS. It mainly focuses on the regulation of the nervous system, BBB, and peripheral immune system, closely follows the pathological mechanism of MS, and explores the treatment of MS by inhibiting neuroinflammation and promoting myelin regeneration. The cross-integration of pharmacology, clinical medicine, pharmaceutics and systems biology based on modern biotechnology for the potential active metabolites in the regulation of immunity and inflammation is the key path to fully tap the therapeutic value of these natural active substances and develop safe and effective candidate therapies for MS.
